# PGPIPN, a Therapeutic Hexapeptide, Suppressed Human Ovarian Cancer Growth by Targeting BCL2

**DOI:** 10.1371/journal.pone.0060701

**Published:** 2013-04-08

**Authors:** Wei Wang, Fang Gu, Cai Wei, Yigui Tang, Xin Zheng, Mingqiang Ren, Yide Qin

**Affiliations:** 1 Department of Biochemistry & Molecular Biology, Anhui Medical University, Heifei, Anhui, China; 2 Georgia Health Sciences University Cancer Center, Augusta, Georgia, United States of America; University of Pittsburgh, United States of America

## Abstract

Bioactive peptides, either derived from nature resources or synthesized by rational design, have been demonstrated potential for therapeutic agents against numerous human diseases, including cancer. However, the mechanism of therapeutic peptides against cancer has not been well elucidated. Here we show that PGPIPN, a hexapeptide derived from bovine β-casein, inhibited the proliferation of human ovarian cancer cells line SKOV_3_ as well as the primary ovarian cancer cells *in vitro*. Consistently, PGPIPIN also decreased tumor growth rate in xenograft ovarian cancer model mice in a dose-dependent manner. Further study demonstrated that the anti-tumor effect of PGPIPN is partially through promoting cell apoptosis by inhibiting BCL2 pathway. Thus, our study suggests that PGPIPN is a potential therapeutic agent for the treatment of ovarian cancer or other types of cancer.

## Introduction

Ovarian cancer is the most lethal gynecological malignancy. The incidence of ovarian cancer is the third in gynecologic cancer after breast and cervix cancer among women, but is the most death tolls in gynecologic cancer. The conventional course of therapy for ovarian cancer includes surgical debulking of the tumor mass followed by adjuvant chemotherapy. Although much progress has been achieved in the development of cancer therapies in recent years, problems continue to arise particularly with respect to chemotherapy due to side-effects, resistance to and low specificity of currently available drugs [1]. Therefore, there is a need to develop safe and effective anti-cancer agents [2].

Peptide therapeutics is a promising field for emerging anti-cancer agents, mainly due to that these peptides can easily obtain either from nature resources or rational design based on the target protein structure. Indeed, several studies have shown that a number of bioactive peptides inhibited tumor cell growth in preclinical trails [3]–[5]. In particular, these therapeutic peptides usually have no or limited toxicity [2]. For example, an anticancer bioactive peptide (ACBP) extracted from goat spleens dramatically inhibited human gastric tumor growth in a xenograft model with no apparent cytotoxicity to host [3]. Subsequent studies suggested that the anticancer effects of some bioactive peptides could be attributed to their abilities in induction of cell apoptosis and cell cycle arrest [3], [6]–[7]. Recent studies have revealed some peptides can impair a specific signaling pathway and subsequently inhibited the tumor growth or metastasis. Such as, a peptide of SAH-BCL9 (stabilized alpha helix of B cell lymphoma 9) targeting beta-catenin inhibited oncogenic Wnt activity, suppressed the growth and metastasis of colorectal cancer and multiple myeloma xenograft, and promoted the tumor cells apoptosis [8]. The hydrocarbon-stapled peptide SAHM1 prevented assembly of the active transcriptional complex of Notch, and consequently inhibited cell proliferation *in vitro* and tumorigenesis in a mouse model of NOTCH1-driven T-cell acute leukemia and lymphoma [9].

In addition to their primary nutritional values, milk proteins are important sources of biologically active peptides [10]–[11]. Milk proteins are the precursors of many biologically active peptides which are inactive in the precursor proteins, but can be released and activated by enzymatic proteolysis [12]. Some peptides derived from milk protein are good candidates for clinical anticancer agents or adjuvant since they are easily absorbed with less potential toxicity. Additionally, here are increasing studies showing that bioactive milk peptides can be absorbed intact from the intestinal lumen into the blood circulation - these may thus serve as novel pharmaceutical agents, which did not cause significant side effects in healthy human [13]. In fact, the exploration of the anti-cancer effects of bioactive peptides from milk proteins emerges as one of the hottest regions recently. For example, talactoferrin (TLF), a recombinant human lactoferrin (LF), is a new developed anticancer agent which has entered phase III clinical trials [14]–[15].

PGPIPN (Pro-Gly-Pro-Ile-Pro-Asn, residues 63–68 of ß-casein), an immunomodulatory peptide, was the discovered active peptide from bovine milk protein [16]–[18]. Previous studies have been shown that this peptide plays an important role in immune defense response. For example, the peptide enhanced phagocytic activity of macrophages *in vitro* against sheep red blood cells (SRBCs) and protected mice against infection with Klebsiella pneumoniae *in vivo*. In recent years, our laboratory dedicated to explore the physiological functions of PGPIPN. Compared with other immunomodulating peptides, PGPIPN is more resistant to the degrading-enzyme system due to its rich proline content [19]. Moreover, a branched-chain amino acid (BCAA) of this peptide helps in some extent to resist microorganisms. Our previous studies suggested that PGPIPN significantly promoted the peritoneal macrophage phagocytosis and the red blood cell immunity in rats. It can also stimulate the proliferation of lymphocyte in both rats and mice [20]–[21]. Moreover, our subsequent study demonstrated that this peptide has good antioxidant effect *in vivo* [22].

Here we show that the hexapeptide PGPIPN can effectively inhibit ovarian cancer cell proliferation, induce cancer cell apoptosis and decrease tumor growth in xenograft ovarian cancer model. The findings in the present study provide the proof of concept for using PGPIPN as a potential therapeutic agent for the treatment of ovarian cancer.

## Materials and Methods

### Reagents

The PGPIPN (the purity was confirmed by RP-HPLC to be >99.5%) was provided by Shanghai Sangon Biological Engineering Technology. Tunel kit was purchased from Roche. Gene Elute Mammalian Genomic DNA Miniprep Kit was purchased from Sigma. Mouse monoclonal antibodies of BCL2, Bax, and β-Actin were purchased from Santa Cruz Biotechnology, Inc.

### Cell Cultures

Human ovarian cancer cell line SKOV_3_ and human normal hepatic cell line LO2 were originally purchased from ATCC. Murine embryo fibroblast cells (MEFs) originally from Harvard Medical School in the United States, *p*53 gene of which had been knocked out, was presented by Professor Hongbing Zhang in Chinese Academy of Medical Sciences & Peking Union Medical College, China. These cell lines were cultured in DMEM with 10% FBS in 5% CO_2_ at 37°C. For primary ovarian cells culture, fresh primary ovarian tumor tissue, which was assessed and classified as serous ovarian adenocarcinoma (I-II grade) according to WHO criteria, were obtained from 5 patients with ovarian cancer at initial debulking surgery in the first affiliated hospital of Anhui Medical University. All patients signed written consents documenting donation of their tissue for research purpose according to the Declaration of Helsinki before tissue deposition. This study was approved by the Anhui Medical University Review Board. The tumor tissues were cut into small pieces about 1.0 mm^3^, and rinsed with PBS two times and digested with 0.25% trypsin in sterile centrifuge tube at 37°C for 30 minutes. To obtain the single suspension cells, the above digested tissues were filtered with 100 um cell strainer. After centrifuged at 1000 rpm for five minutes, the cell pellet was re-suspended in DMEM medium supplementary with 10% human serum. When the cells grew to 70–80% confluent, the culture medium in flask was drained; the cells were digested with 0.25% collagenase II. When approximately 1/3 cells falling down by observing under a microscope, digestion was immediately stopped and the culture medium in flask was drained again. Owing to their shedding first, the most of the fibroblasts were eliminated by collagenase digestion. The remained cells were cultured continually for cell proliferation assay. The portion of these cells were made to the cell slide and identified by using immunofluorescence of cytokeratin 7 to assay their purity.

### Cell Proliferation Assay

SKOV_3_ cells were seeded into 96-well plates in octuplicate at a starting density of 5×10^3^ cells/well. After overnight culture, PGPIPN was added at the final concentrations of 0 (as control), 3×10^−8^, 3×10^−7^, 3×10^−6^, 3×10^−5^, 3×10^−4^, 3×10^−3^ and 3×10^−2^ g/L, respectively. 5-Fluorouracil (5-FU) at 3×10^−3^ g/L was added in the same plate as positive control. The proliferation of the cells was measured at different time point by the MTT method, as described [23]. The following formula was used to calculate the cell growth inhibition ratio (IR): IR (%) = (1 - the experimental group A_490 nm_ value/control group A_490 nm_ value) × 100%. Each experiment was triplicated independently.

Using the same procedure, the growth inhibition of PGPIPN on primary ovarian cancer cells were also assayed, except for the final concentrations of PGPIPN at 0 (as control), 3×10^−6^, 3×10^−5^, 3×10^−4^, 3×10^−3^ and 3×10^−2^ g/L, respectively. The experiments were duplicated with primary ovarian cancer cells from five patients, respectively.

For the detecting the toxicity of PGPIPN, the growth inhibitions of PGPIPN on untransformed cell lines LO2 and MEFs were assayed with the same procedure as that of SKOV_3_ cells, except for the final concentrations of PGPIPN at 0 (as control), 3×10^−4^, 3×10^−3^, 3×10^−2^, 3×10^−1^ and 3 g/L, respectively. Each experiment was triplicated independently.

### Morphological Observation of Cells Treated with PGPIPN

The dynamic morphological changes of the SKOV_3_ cells treated with PGPIPN were observed with optical microscope. The glass cover of crawling cell was prepared. The cover glass nearly full of cell on its surface was taken for H&E staining, with procedure according to reference [24]–[25]. The method used to observe the apoptosis of SKOV_3_ cells with transmission electron microscope has been described previously [26].

### Detection of Apoptosis in Cultured Cells by FCM

Apoptotic cells were detected using FITC-conjugated Annexin-V and propidium iodide (PI) from Sigma. Cells were washed twice with cold PBS and resuspended in Annexin-V binding buffer (10 mM HEPES, 140 mM NaCl and 5 mM CaCl_2_) at a concentration of 1×10^6^ cells/mL. Then single suspension of 1×10^6^ SKOV_3_ cells was prepared in a 5 mL culture tube according to the reference [23], in which 5µL Annexin-V-FITC at 10 ug/mL and 10 µL propidium iodide at 10 ug/mL was added. Then the tube was gently vortexed and incubated for 15 min at room temperature in the dark. Binding buffer (400 µL) was then added to each tube and the cells were analyzed by flow cytometry.

### Animal Treatment

Twenty-four healthy female nude mice were used in the researches. All animal experiments were carried out under the protocol approved by the Institutional Animal Care and Use Committee of the Anhui Medical University. During animal experiments, as far as possible animal suffering was ameliorated. All nude mice were euthanized at the end of experiments. The nude mice in inbred strain (BALB/cAnN-nu/nu), 8–10 weeks old, were purchased from Shanghai Slac Laboratory Animal Co. Ltd. All mice were kept in SPF-class sterile room in the Anhui Provincial Center for Medical Experimental Animals.

Each nude mouse was inoculated subcutaneously in its right armpit with 0.2 ml SKOV_3_ cells suspension at (1×10^7^) cells/ml. On the second day after inoculation, the mice were randomly divided into four groups: NS (normal saline), low dose PGPIPN, high dose PGPIPN and 5-FU (as positive control) groups. NS, low dose PGPIPN, high dose PGPIPN and 5-FU groups were intraperitoneally injected with 0.2 mL saline, 0.2 mL PGPIPN at 0.25 g.L^−1^, 0.2 mL PGPIPN at 0.50 g.L^−1^ and 0.2 ml 5-FU at 30 mg/kg body weight, respectively. The drugs were given once every other day for 4 weeks.

The tumor size was measured by with vernier caliper weekly, and calculated according to the formula as follow: V = (1/2) ab^2^, where V = tumor volume; a = the largest diameter of tumor; b = the most trails of tumor. At the fourth weekend after planting, all nude mice were euthanized, and xenograft tumors were weighted. The xenograft tumors were frozen in liquid nitrogen for subsequent experiments.

### TUNEL (TdT-mediated dUTP Nick-End Labeling) Assay

The tumor specimens were fixed and embedded with paraffin. The TUNEL assay was performed as the manufacture’s manual (ROCHE). Finally, the sections were counterstained with hematoxylin. Apoptotic cells were quantified by light microscopy on hematoxylin and eosin (HE) stained sections by averaging the number of cells with homogeneously dense chromatin or karyorrhectic nuclear fragments in photographs of five randomly selected ×40 fields. The cases were evaluated by two independent examiners. Apoptosis index (AI) was calculated as following formula: AI = (the number apoptotic cell/the total number of cells) × 100%.

### Fragmentation Assay of DNA by Agarose Gel Electrophoresis

DNA fragments in the tumor tissues were assayed by agarose gel electrophoresis, according to the method described by Sambrook & Russell (2001) [27]. DNA in the tumor tissues was extracted using Gene Elute Mammalian Genomic DNA Miniprep Kit (Sigma), and subjected to electrophoresis in 1.5% agarose gel (containing 0.25 µg/ml ethidium bromide). The electrophoretic bands were visualized and photographed under transmitted ultraviolet light.

### Western Blot Analysis of BCL2 and Bax Proteins in the Tumor Tissues

The proteins were isolated from the xengrafted tumor samples and were separated by SDS-PAGE using the standard protocol. After blocked with 5% (w/v) dry skim milk, membranes were incubated with primary antibodies (mouse monoclonal Bax, BCL2 and β-Actin antibodies, 1∶1000 dilution) according to the manufacturer’s instructions (Santa Cruz Biotechnology) and then incubated with horseradish peroxidase conjugated secondary antibody (goat anti-mouse IgG, 1∶8000 dilution). The proteins were detected with the enhanced chemiluminescence (ECL) system (Pierce, Rockford, IL) followed by exposure to X-ray film. β-Actin was used as a loading control. Digital images were captured by Gel Doc™ gel documentation system (Bio-Rad, USA) and intensities were quantified using Quantity-One software version 4.62 (Bio-Rad, USA).

### Statistical Analysis

All measured data were presented as mean ± *SD*. The differences among groups were analyzed using the one-way ANOVA by SPSS12.0 statistical software. Statistical significance was defined as *P*<0.05.

## Results

### PGPIPN Treatment Induced Cell Proliferation Inhibition and Apoptosis of SKOV_3_ Ovarian Cancer Cells *in vitro*

**Figure 1:**
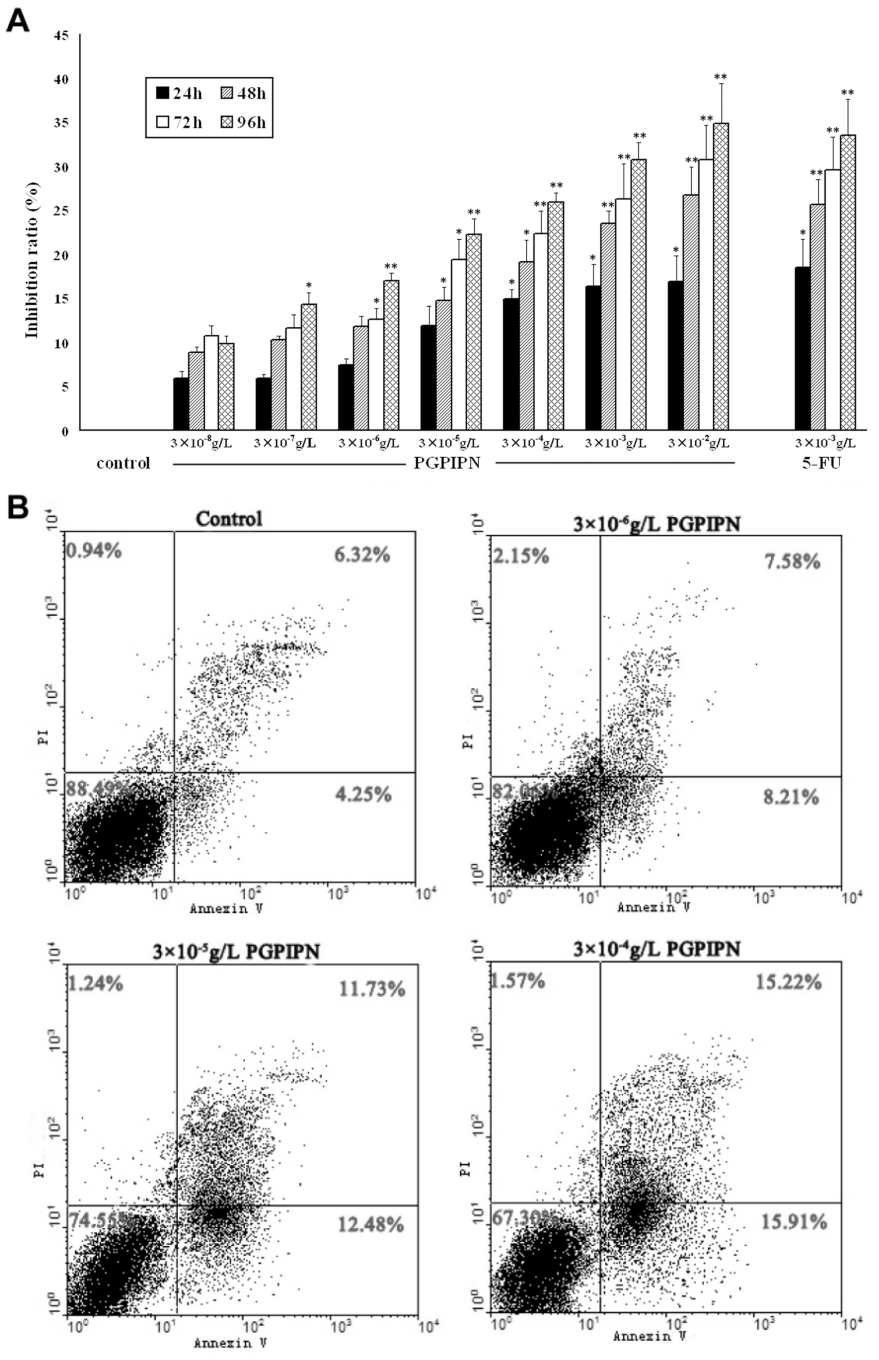
PGPIPN induces human ovarian cancer SKOV_3_ cells underwent growth inhibition and cell apoptosis. (**A**) PGPIPN at different concentrations inhibits the proliferation of SKOV_3_ cells, measured at different time points. Data shown are mean±*SD* of three independent experiments,*P<0.05, **P<0.01 compared with control (the vehicle group). (**B**) Flow cytometry analysis shows that PGPIPN treatment induced SKOV_3_ cells apoptosis after 48 h drug exposure. This measurement was biologically triplicated.

PGPIPN has been shown to play an important role in immunomodulatory therapy and other effects in many researches [19]–[22], [28]–[29]. This intrigues us to investigate whether PGPIPN can be used as anticancer agent. For this end we first investigated the effect of PGPIPN on the proliferation of SKOV_3_ cells. To our surprise, PGPIPN can effectively suppress the SKOV_3_ cells growth even at low dosage of 3×10^8^ g/L (Figure 1A). This inhibition capacity of PGPIPN was compared with 5-FU treatment when the cells were exposed to high concentration of 3×10^3^ g/L. The inhibition effect of PGPIPN also showed time- and dose-dependent manor. Furthermore, compared with the control, PGPIPN treatment led to obvious morphological changes in SKOV_3_ cells, including cell shrinking, karyopyknosis, and appearance of the cytoplasmic vacuoles in some cells (date not shown). There also showed a deeply stained in the nuclear section and a great amount of cytoplasmic bodies or small pieces in the PGPIPN-treated cells, which are the typical characteristics of apoptic cells (data not shown). To validate this observation, we performed the apoptosis assay with Annexin V-TITC and PI double-staining method. PFPIPN treatment clearly induced SKOV_3_ cells underwent apoptosis after 48 h drug exposure at different concentrations (Figure 1B).

### PGPIPN can Effectively Inhibit Human Primary Ovarian Cancer Cell Growth *in vitro*

**Figure 2:**
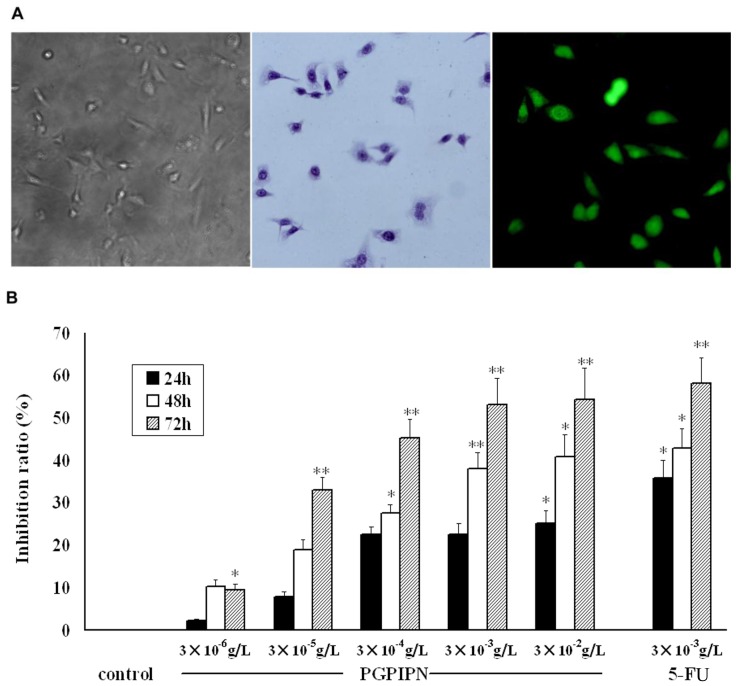
PGPIPN suppressed human primary ovarian cancer cells growth. (A) A represent morphology of ovarian carcinoma cells from a patient growing in the primary culture medium (×100, left panel), H&E stained (middle panel) and anti-cytokeratin 7-FITC stained (right panel). (B) Cell proliferation assay shows that PGPIPN at different concentrations suppressed primary ovarian cells growth. Data are calculated from 5 primary cancer cells measurements and presented as mean, and error bars refer to SD of decuplicate analyses, *P<0.05, **P<0.01 compared with control (the vehicle group).

Next, we further test whether PGPIPN can also inhibit the human primary cancer cells growth. We successfully insolated and established 5 primary cancer cells from 5 patients with ovarian cancer at initial debulking surgery in the first affiliated hospital of Anhui medical university. These primary cells were culture in our laboratory. These cell are morphologically presented as typical epithelium cells (Figure 2A left and middle panel). These primary ovarian cancer cells were further identified by immunocytochemistry assay with anti-cytokeratin 7 staining (Figure 2A right panel). The average purity of ovarian carcinoma cells was approximately 85% based on cytokeratin 7 staining. To investigate whether PGPIPN can decrease growth of primary ovarian cancer cells, we seeded these cells in 96-well plates. After over-night growth these cells were treated with PGPIPN at different concentrations for 24, 48 and 72 h. As shown in Figure 2B, treatment with different concentrations of PGPIPN led to a significant inhibition of ovarian carcinoma cell proliferation, and the inhibition effect showed time- and dose-dependent manor. All these indicate that the primary ovarian cancer cells are also sensitive to PGPIPN treatment.

### PGPIPN had Little or no Effect on Untransformed Cell Growth *in vitro*

**Figure 3:**
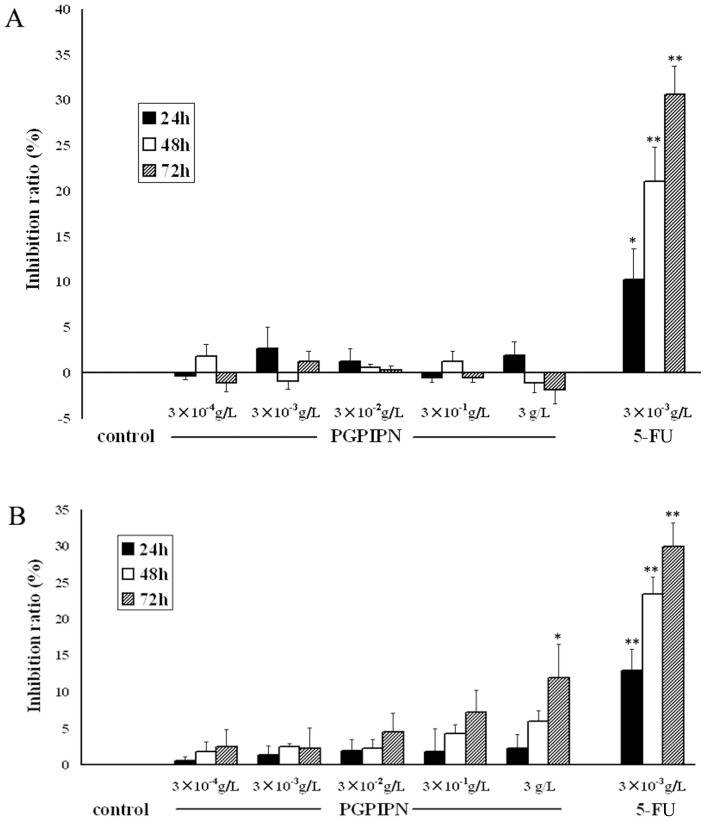
PGPIPN had little or no effect on untransformed cell growth *in vitro*. (A) PGPIPN had no effect on the proliferation of LO2 cells. (B) PGPIPN slightly affected the proliferation of MEFs, which was significantly inhibited only at a high dose (0.3 g/L ) of the peptide for 72 h. Results are expressed as mean ± *SD* from three independent experiments, *P<0.05, **P<0.01 compared with control (the vehicle group).

The cytotoxicities of PGPIPN towards untransformed cell lines were investigated. MTT assay was performed to assay the effects of PGPIPN on the proliferations of human normal hepatic cell line LO2 and murine embryo fibroblast cells (MEFs). The peptide was found to have no effect on the proliferation of LO2 cells (Figure 3A). The proliferation of MEFs was slightly affected by PGPIPN, which was significantly inhibited only at a high dose (0.3 g/L ) of the peptide for 72 hours, but the influence was much smaller compared with positive control group (5-FU group) (Figure 3B). Consequently, PGPIPN exhibited little or no cytotoxicity towards untransformed cell, as compared with the traditional anticancer drugs (5-FU).

### PGPIPN Significantly Decreased Xenografted Tumor Growth *in vivo*

**Figure 4:**
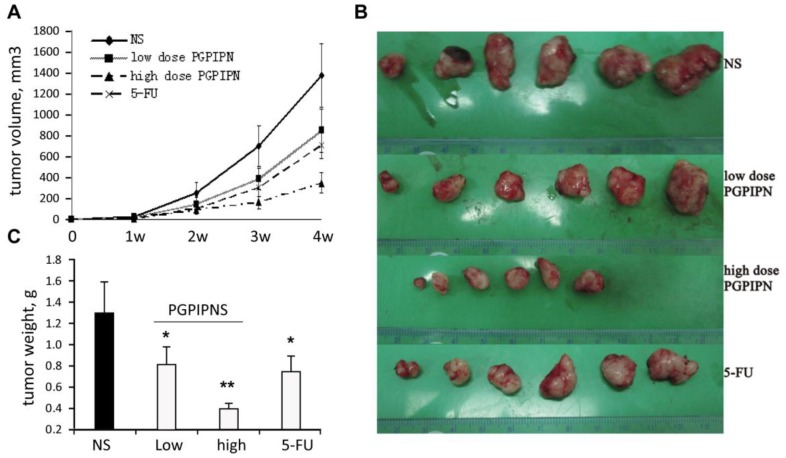
PGPIPN significantly decreased xenografted tumor growth *in vivo*. PGPIPN remarkably inhibited tumor growth after 4-weeks treatment (A) and decreased the tumor size (B) and tumor weight (C) at the end of treatment. Data are presented as mean ± *SD* of 6 mice, *P<0.05, **P<0.01 compared with NS group.

To determine whether PGPIPN has an anti-tumor effect *in vivo*, we engrafted SKOV_3_ cells subcutaneously into nude mice. Twenty-four mice were randomly divided into four groups: NS (normal saline), low dose PGPIPN, high dose PGPIPN and 5-FU (as positive control) groups as described in Materials and Methods. PGPIPN was administered intraperitoneally every other day beginning from the second day after inoculation of tumor cells. Saline served as a negative control, and 5-fluorouracil was used as a positive control. These mice were treated for 4 weeks. At the fourth weekend, tumors were removed and measured. Both dosages of PGPIPN can significantly inhibit tumor growth compared to the NS group (Figure 4A). Tumors in the NS group grew to an average volume of (1370.25±303.12) mm^3^. In contrast, tumors in the PGPIPN low-dose group, PGPIPN high-dose group and 5-FU group grew to an average volume of (845.43±205.09) mm^3^, (346.78±97.16) mm^3^ and (705.82±124.47) mm^3^, respectively (Figure 4A). Compared with the NS group, the inhibitory rates in PGPIPN low-dose group, PGPIPN high-dose group and 5-FU group were 36.92%, 68.46% and 41.54% respectively. Consistently, the tumor sizes (Figure 4B) or weights (Figure 4C) were remarkably decreased in all drugs treatment groups as compared with control group. Together these data indicate that PGPIPN can effectively inhibit xenografted tumor growth *in vivo*, which is in comparison to the traditional anti-ovarian drug, 5-fluorouracil.

### Tumor Growth Inhibition Induced by PGPIPN is Associated with Cell Apoptosis *in vivo*

**Figure 5:**
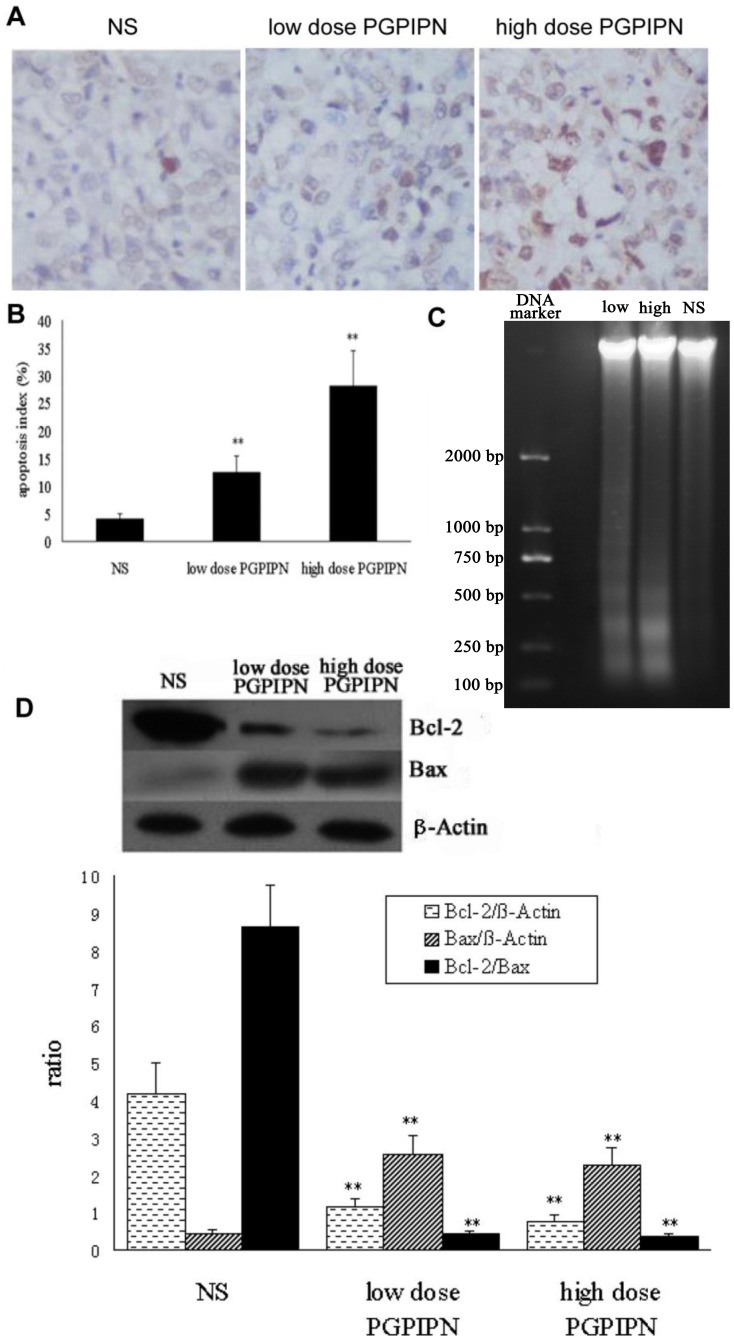
PGPIPN induced tumor growth inhibition associated with cell apoptosis. (A) TUNEL assay shows the apoptotic tumor cells in PGPIPN-treated samples extracted from xenograft mice (×400). (B) Apoptotic index was calculated as following formula: AI = (the number apoptotic cell/the total number of cells )× 100% (mean ± *SD*,n = 6), **P<0.01. (C) DNA fragment assay shows that PGPIPN induced tumor DNA degradation in high dose PGPIPN group (high) and low dose PGPIPN group (low), but not in normal saline group (NS). (D) Protein levels of BCL2 and Bax were examined by western blot in tumor samples extracted from xenografted mice (top panel). The band intensities were measured by Imaging J software and summarized (bottom panel). The data are from 6 tumors of each group, *P<0.05, **P<0.01 compared with NS group.

Flow cytometry analysis showed that PGPIPN-induced SKOV_3_ cells underwent apoptosis *in vitro* (Figure 1B). To test whether PGPIPN can also induce cell apoptosis in tumor treatment *in vivo*, we performed TUNEL assay on the tumor samples extracted from engrafted nude mice. The number of TUNEL-positive cells in tumor samples extracted from the PGPIPN treatment groups was significantly increased (Figure 5 A and B). In consistent with this observation, DNA fragment assay also demonstrated the remarkable DNA degradation in PGPIPN-treated tumors samples, indicating the high percentage apoptotic cells in these samples (Figure 5 C). In contrast, NS treated tumor samples did not show this typical DNA ladder pattern in electrophoresis (Figure 5 C). Taken together, these results suggest that PGPIPN inhibits the tumor growth through induction of tumor cells undergoing apoptosis.

To further confirm that induction of apoptosis is involved in the regression of tumor growth after PGPIPN treatment, two apoptosis-related genes, *BCL2* and *bax*, were evaluated via western blotting analysis. Immunoblot assay with BCL2 and Bax antibodies demonstrated that BCL2 protein levels were significantly reduced in PGPIPN-treated groups (*P*<0.05 or *P*<0.01) compared with that from the control group (Figure 5D). In contrast, the expressions of Bax were dramatically up-regulated in PGPIPN-treated groups, as compared with the control (Figure 5D). These data clearly suggest that PGPIPN inhibited tumor growth at least in part through inducing cell apoptosis.

## Discussion

Many bioactive peptides derived from milk protein are inactive within their parent milk proteins, and upon released during digestion or food processing, they may act as regulatory compounds with biologic activities. In the present study, we are the first to show that PGPIPN can profoundly inhibit human ovarian cancer cells both in xenograft mouse model as well as in the primary cancer cells without non-specific toxic effects, suggesting PGPIPN may be a novel anticancer agent and should be considered for further preclinical trial in other cancer types.

The therapeutic peptides may through different mechanisms undergo the anticancer effects dependent on their characteristics. Some peptides interact very specifically with cyclins and/or cyclin-dependent kinases or with members of apoptotic cascades [30]–[31]. Recent studies have shown that the peptides can impair the specific signaling pathways and subsequently inhibited the tumor growth or metastasis [8]–[9], [32]. In our study, the antiovarian cancer of PGPIPN was mainly through the induction of apoptosis mediated by down-regulation of BCL2 (Figure 5). In consistent with this observation, we also found many morphological changes related to cell apoptosis, such as, cell spikes, surface blisters, blebs and cellular rounding and nuclear disintegration (date not shown).

Exactly how PGPIPN down-regulated BCL2 expression is yet to be determined. Earlier researches showed that the two peptides derived from human β-casein (GLF and VEPIPY), can bind to the cell membrane [33]–[34]. Recent some researches indicated that bioactive peptides derived from bovine milk proteins were capable of binding and affecting cells [13], [35]–[38]. For example, Kreider RB et al. [13] reported that a novel milk peptide mixture inhibited the tyrosine kinase activity of epidermal growth factor receptor (EGFR), vascular endothelial growth factor receptor 2 (VEGFR2), and insulin receptor (IR) respectively. This multi-kinase inhibitor caused apoptosis in HT-29 colon cancer cells *in vitro*, whereas the milk peptides mixture was safe to consume orally for healthy volunteers and no clinically significant side effects were reported [13]. Fiedorowicz E, et al. reported the bioactive peptides-casomorphin-7 (YPFPGPI), casoxin-D (YVPFPPF) and casoxin-6 (SRYPSY) from bovine caseins could bind μ-opioid receptor on cytomembrane to influence the proliferation and cytokine secretion of human peripheral blood mononuclear cells (PBMCs) [35]. Almansour NM et al. [2], [39] reported that myxoma virus peptide analogue can target the Akt signaling pathway as a possible cell death pathway in human skin cancer cells.

In our ongoing experiment, we observed that PGPIPN labeled with fluorescein isothiocyanate (FITC) emerged on SKOV_3_ cell membrane under confocal laser scanning microscope (CLSM) (date not shown), indicating this peptide could bind to cell membrane, like some other peptides from milk protein. We reasoned that PGPIPN may bind to cell specific receptor(s) and dysregulate cellular signal transduction pathways, and reduced the BCL expression, then induced cell apoptosis. All these need to be investigated in the future work. Of course, PGPIPN may through via other mechanisms to affect cell growth.

In summary, the hexapeptide PGPIPN can effectively inhibited ovarian cancer cell proliferation both in vitro and in vivo. This inhibition effect is mainly through PGPIPN induced cancer cell apoptosis. These data suggest that PGPIPN is a potent therapeutic peptide for the treatment of ovarian cancer, and provide a rationale for further evaluation in clinic.
